# Diversity, compositional and functional differences between gut microbiota of children and adults

**DOI:** 10.1038/s41598-020-57734-z

**Published:** 2020-01-23

**Authors:** Djawad Radjabzadeh, Cindy G. Boer, Sanne A. Beth, Pelle van der Wal, Jessica C. Kiefte-De Jong, Michelle A. E. Jansen, Sergey R. Konstantinov, Maikel P. Peppelenbosch, John P. Hays, Vincent W. V. Jaddoe, M. Arfan Ikram, Fernando Rivadeneira, Joyce B. J. van Meurs, André G. Uitterlinden, Carolina Medina-Gomez, Henriette A. Moll, Robert Kraaij

**Affiliations:** 1 Department of Internal Medicine, Erasmus MC, Rotterdam, The Netherlands; 2 Department of Paediatrics, Erasmus MC, Rotterdam, The Netherlands; 3 The Generation R Study, Erasmus MC, Rotterdam, The Netherlands; 4 Department of Gastroenterology and Hepatology, Erasmus MC, Rotterdam, The Netherlands; 5 Department of Medical Microbiology and Infectious Diseases, Erasmus MC, Rotterdam, The Netherlands; 6 Department of Epidemiology, Erasmus MC, Rotterdam, The Netherlands; 7 Department of Public Health and Primary Care/LUMC Campus The Hague, Leiden University Medical Centre, Leiden, The Netherlands

**Keywords:** High-throughput screening, Predictive markers

## Abstract

The gut microbiota has been shown to play diverse roles in human health and disease although the underlying mechanisms have not yet been fully elucidated. Large cohort studies can provide further understanding into inter-individual differences, with more precise characterization of the pathways by which the gut microbiota influences human physiology and disease processes. Here, we aimed to profile the stool microbiome of children and adults from two population-based cohort studies, comprising 2,111 children in the age-range of 9 to 12 years (the Generation R Study) and 1,427 adult individuals in the range of 46 to 88 years of age (the Rotterdam Study). For the two cohorts, 16S rRNA gene profile datasets derived from the Dutch population were generated. The comparison of the two cohorts showed that children had significantly lower gut microbiome diversity. Furthermore, we observed higher relative abundances of genus *Bacteroides* in children and higher relative abundances of genus *Blautia* in adults. Predicted functional metagenome analysis showed an overrepresentation of the glycan degradation pathways, riboflavin (vitamin B2), pyridoxine (vitamin B6) and folate (vitamin B9) biosynthesis pathways in children. In contrast, the gut microbiome of adults showed higher abundances of carbohydrate metabolism pathways, beta-lactam resistance, thiamine (vitamin B1) and pantothenic (vitamin B5) biosynthesis pathways. A predominance of catabolic pathways in children (valine, leucine and isoleucine degradation) as compared to biosynthetic pathways in adults (valine, leucine and isoleucine biosynthesis) suggests a functional microbiome switch to the latter in adult individuals. Overall, we identified compositional and functional differences in gut microbiome between children and adults in a population-based setting. These microbiome profiles can serve as reference for future studies on specific human disease susceptibility in childhood, adulthood and specific diseased populations.

## Introduction

The human gut microbiome is dynamic, shaped by multiple factors and has been shown to play an important role in human health. Several studies have reported an association between alterations in the composition of the gut microbiome and various gastrointestinal (GI)^1–9^ and non-GI^10–15^ disease conditions in both children and adults. These changes in composition of the microbiome (known as dysbiosis) and their associations with health and disease have led to an increased interest in the complex microbial community of the gut^16,17^. However, our understanding of the relationship between the gut microbiome and health and disease still remains superficial at best. A fundamental issue hampering progress in this respect is that our lack of knowledge on the composition and variation of the gut microbiome across the human lifespan remains limited. So far, most microbiome studies have been relatively small in terms of sample size and have usually focused on specific diseases and phenotypes, with most publications having used a case-control study design, what makes the analyses more vulnerable to methodological challenges. A better understanding of the variation in microbiome composition (from stool or other sources) and its role in the etiology of chronic diseases can be achieved through the study of large and well-characterized population-based cohorts, which generate rich information on many different physiological parameters, disease status, medication use, dietary intake, and other layers of “omics” data from individual participants.

Up to now, microbiome studies have focused mainly on adult populations, whereas it has been shown that the intestinal microbiome undergoes dynamic changes in diversity and composition during the human lifespan and particularly during development; with the most substantial changes believed to occur throughout childhood^18–20^. Interestingly, emerging data suggest that early alterations in the gut microbiome are associated with an increased risk of developing diseases later in childhood and adulthood e.g., asthma^21,22^ and Crohn’s disease^23^. These studies were, however, limited by low sample sizes. If the field of microbiota dynamics is to move forward, it requires studies in well-characterized general population cohorts of different ages, including studies investigating pediatric cohorts. So far, a complete description of diversity, compositional and functional differences between children and adults in a large, homogeneous and population-based cohorts have not been reported.

The above-mentioned considerations prompted us to profile the stool microbiome composition of children and adults within two large, independent and extensively characterized population-based cohorts: the Generation R Study (GenR, visit at 9 years) and the Rotterdam Study (RS, sub-cohort RSIII-2). In this publication, we report our findings on the differences in gut microbiota between 2,111 children aged 9-12 years and 1,427 adults >40 years of age living in the same city with similar urban surroundings.

## Methods

### Study populations and sample collection

The Generation R Study (GenR) is a population-based prospective multi-ethnic pregnancy cohort study from fetal life until young adulthood conducted in the city of Rotterdam^24^. The study was designed to identify early environmental and genetic factors and causal pathways underlying normal and abnormal growth during development during childhood. GenR recruited 9,749 children undergoing several rounds of follow-up after birth. Stool sample collection started in 2012 at a mean age of 9.8 years (SD: 0.32). Ethics approval was obtained from the Medical Ethical Committee of Erasmus MC (MEC-2012-165) and written informed consent was obtained from all participants’ parents. All methods were performed in accordance with the Declaration of Helsinki.

The Rotterdam Study (RS) is a prospective population-based cohort study established in 1990 to study determinants of disease and disability in Dutch adult individuals. The original design and updates of this study have been described in detail^25^. RS consists of four sub-cohorts and comprises approximately 18,000 inhabitants of the Ommoord suburb in Rotterdam (which is predominantly populated by individuals of European ethnicity (about 96%)), aged ≥40 years. The collection of fecal samples started in 2012 among the RS-III sub-cohort comprising 3,932 participants. This study was approved by the Medical Ethical Committee of Erasmus MC (MEC-02-1015) and by the Ministry of Health, Welfare and Sport of the Netherlands. All subjects provided written consent prior to participation in the study. All methods were performed in accordance with the Declaration of Helsinki.

Stool samples were collected at home by the participants using a Commode Specimen Collection System (Covidien, Mansfield, MA). An aliquot of approximately 1 g was transferred to a 25 × 76 mm feces collection tube (Minigrip Nederland, Lelystad, The Netherlands) without preserving agent included and sent through regular mail to the Erasmus MC. A short questionnaire addressing date and time of defecation, current or recent antibiotics use (past year), recent probiotics use (past 3 days), and recent travel activities (past month), was filled out by the participants and included in the package. Upon arrival at Erasmus MC, samples were recorded and stored at −20 °C. The only modification in the collection protocol for the GenR cohort (as compared to RS) was that, in case of delay, the samples were stored by participants at 4 °C (home fridge) before mailing to Erasmus MC. This modification allowed to better preserve samples that were produced in the evening or during the weekend.

### DNA isolation

Stool samples from the two cohorts were randomly taken from the −20 °C freezer and allowed to thaw for 10 minutes at room temperature prior to DNA isolation. Samples with inconsistent or lack of information on sample production and samples in which mold growth was observed were excluded (Supp. Fig. 1). An aliquot of approximately 300 mg was homogenized in stool stabilizing buffer according to the manufacturer’s protocol (Arrow Stool DNA; Isogen Life Science, De Meern, The Netherlands). Homogenized samples were bead beated in Lysing Matrix B tubes containing 0.1 mm silica beads (MP Biomedicals, LLC, Bio Connect Life Sciences BV, Huissen, The Netherlands) using the MagNA Lyser instrument (Roche Diagnostics, Almere, The Netherlands) at 7,000 rpm for 45 seconds. Samples were then centrifuged at 6,000 × g for 5 min and 0.5 ml of supernatant was subjected to automated DNA isolation (Arrow; DiaSorin S.P.A., Saluggia, Italy) according to the manufacturer’s protocol using setting ‘Stool DNA 2.0’ in batches of 12 samples per run. DNA concentration was measured using Quant-iT PicoGreen dsDNA Assay Kit (Thermo Fisher Scientific, Waltham, MA) and DNA was stored at −20 °C.

### 16S rRNA gene sequencing

The V3 and V4 variable regions of the 16 S rRNA gene were amplified using the 309F-806R primer pair and dual indexing (12 base pairs (bp) each on the forward and reverse primers) as previously described^26^. Amplicons were normalized using the SequalPrep Normalization Plate kit (Thermo Fischer Scientific) and pooled. The pools were purified prior to sequencing using Agencourt AMPure XP (Beckman Coulter Life Science, Indianapolis, IN) and the amplicon size and quantity of the pools were assessed on the LabChip GX (PerkinElmer Inc., Groningen, The Netherlands). PhiX Control v3 library (Illumina Inc., San Diego, CA) was spiked into (~10%) the pooled amplicon libraries and each pool was sequenced on an Illumina MiSeq sequencer (MiSeq Reagent Kit v3, 2 × 300 bp) at an average depth of 50,000 read-pairs per sample.

### Data pre-processing, OTU picking and quality control

Phylogenetic multi-sample profiling was performed using an *in-house* developed analysis pipeline (microRapTor) based on QIIME (version 1.9.0)^27^ and UPARSE (version 8.1)^28^ software packages. Briefly, index sequences (12 bp) were removed from each read and concatenated to generate a unique index of 24 bp for each read-pair. Spacer and primer sequences were removed using TAGCleaner (version 0.16)^29^. Paired reads were merged using PEAR (version 0.9.6)^30^ with the following settings: minimum overlap of 10 bp (default) and an average read quality phred-score of 20 over a 30 bp sliding window. Merged reads shorter than 200 bp were discarded. Reads were de-multiplexed using QIIME including extra quality filtering steps: merged reads were truncated before three consecutive low-quality bases, ambiguous bases were not allowed. Chimeric reads were removed using UCHIME (version 8.1)^28^. Duplicate samples, samples with less than 10,000 reads, and samples from participants that have used antibiotics (self-reported) in one year prior to sample production were excluded (Supp. Fig. 1). The 16 S sequence reads of the remaining samples (2,214 for GenR and 1,544 for RS) were randomly subsampled at 10,000 reads per sample (after rarefaction analysis). Combined reads of all samples, in each cohort separately, were clustered into operational taxonomic units (OTUs) using UPARSE at a minimum cluster identity of 97%. The representative read from each OTU was then mapped to the SILVA rRNA database version 128^31^ using RDP Naïve Bayesian Classifier version 2.12^32^. OTUs containing less than 40 reads were removed as described by Benson *et al*.^33^. This threshold was established based on the correlation analysis of OTU tables of 5 pairs of technical replicates, of which DNA was amplified, sequenced and profiled twice (Supp. Fig. 2). The sequence data was then analyzed for α-diversity metrics (Shannon diversity Index, species richness and Inverse Simpson Index). Final OTU filtering was performed by removing OTUs with a total read count less than 0.005% of all reads and OTUs observed in less than 1% of the total number of samples of each cohort as described previously^34^. The final OTU table was divided into 5 sub-tables at different taxonomic levels (in QIIME environment): phylum, class, order, family, and genus.

### Statistical analyses

All statistical analyses were performed in R^35^ using *vegan*^36^, *phyloseq*^37^ and MaAsLin^38^ packages. As MaAsLin performs paralleled multiple analyses, q < 0.05 (false discovery rate (FDR) multiple testing corrected) was used as significance threshold. All MaAsLin models were adjusted for technical covariates and other confounders as described in the sections below.

### Technical covariates in the initial stool 16S datasets of the two cohorts

After generating the initial 16S datasets of both the RS and GenR cohorts, the effects of collection time (time in mail; TIM) and the yield of DNA isolation runs (Batch; Supp. Fig. 3) on the 16S profiles were assessed. To identify the impact of these technical covariates on the α-diversity and overall profiles, Shannon α-diversity and Bray-Curtis dissimilarity metrics were calculated in each cohort using ‘*diversity’* and ‘*vegdist’* functions in *vegan*, respectively. The effects of the covariates on α-diversity were assessed by linear regression ‘*lm*’ function in package stats. The basic model was adjusted for age and sex. Each of the two technical covariates were stepwise added in order to identify its contribution to the model. Then, likelihood ratio test was used to compare these nested models. Additionally, in GenR self-reported ethnicity was added to the model to inspect and compare the effect of this covariate. Subsequently, permutation analysis of variance (PERMANOVA) was performed to inspect the global effects of these technical covariates on the overall profiles using the ‘*adonis*’ function in vegan. Furthermore, for TIM, additive general linear regression analyses of single genus-level OTUs were performed in MaAsLin. For this, we used sub-sampled datasets that included only samples up to a certain TIM (up to 7 days). Regression analyses in MaAsLin were performed after arcsine square root transformation of the relative abundance data and were adjusted for age, sex and BMI to assess the effect of TIM exclusively.

### Validation of the final stool 16S datasets of GenR and RS

Final datasets were constructed by excluding samples that had been in the mail for 3 (in RS) or 5 (for GenR) days (see Results section). Average relative abundances of the six major phyla were compared to those of other large cohorts. GenR profiles were compared to those reported by the Copenhagen Prospective Study on Asthma in Childhood (COPSAC) cohort (Copenhagen, Denmark; n = 156; age range = 4–6 years^39^), and RS profiles were compared to those reported by the Dutch LifeLines-DEEP (LLD) cohort (Groningen, the Netherlands; n = 1,135; age range = 20–90 years^40,41^) and the Belgian Flemish Gut Flora Project (FGFP) cohort (Leuven, Belgium; n = 1,106; age range = 20–80 years^42^). In addition, we performed association analyses of BMI with Shannon α-diversity and single OTUs at genus level to confirm this well-established association^11,13,40^ in our cohorts, and by comparing associated OTUs observed in our cohorts to those reported by the above-mentioned cohorts.

The LifeLines DEEP (LLD) study was approved by the ethics committee of the University Medical Centre Groningen, document number METC UMCG LLD: M12.113965 and all methods were performed in accordance with the Declaration of Helsinki. All participants signed an informed consent form prior to study enrolment.

Flemish Gut Flora Project (FGFP) procedures were approved by the medical ethics committee of the University of Brussels/Brussels University Hospital (approval 143201215505, 5/12/2012) and all methods were performed in accordance with the Declaration of Helsinki. A declaration concerning the FGFP privacy policy was submitted to the Belgian Commission for the Protection of Privacy. Participants were recruited through repeated announcements in print, audiovisual, and social media as well as through the FGFP website, where volunteers could enroll from January 2013 onwards (http://www.vib.be/nl/mens-en-gezondheid/darmflora-project/Pages/default.aspx).

The Copenhagen Prospective Study on Asthma in Childhood (COPSAC) was approved by all relevant authorities including the Danish Ethical Committee (H-B-2008-093), and the Danish Data Protection Agency (2015-41-3696) and all methods were performed in accordance with the Declaration of Helsinki. Both parents provided informed consent prior to participation.

### Comparing the gut microbiome profiles and functions between children and adults

To compare gut microbiome profiles between children and adults, we selected samples from European participants. Ethnic background of GenR participants was assessed based on self-reported country of birth of four grandparents as described elsewhere^43^. Ethnic background of RS participants was assessed based on self-reported ethnic backgrounds of the four grandparents. We selected European participants by combining all European countries together with Americans, Oceanics and North Africans as evaluated elsewhere^44^. In short, the participant was deemed to be of non-Dutch origin if one parent was born abroad. If both parents were born abroad, the country of birth of the participant’s mother defined the ethnic background. Ethnicities of the parents were derived from the grandparents using the same protocol. The different ethnicities based on the parents country of birth or ethnic background were narrowed to three main ancestry groups: Europeans, including all European countries, together with Americans, Oceanics, and North Africans (with parents from Algeria, Egypt, Libya, Morocco, Sudan, Tunisia, and Western Sahara); Africans, including Sub‐Saharan Africans, Dutch Antilleans, and Surinamese Creoles; and Asians, including all Asian countries and Surinamese Hindustanis. Reads were subsampled at 10,000 reads per sample and pooled. Classification was performed (as described above) in one combined run. Shannon α-diversities were calculated and tested for significant differences between the two cohorts using Wilcoxon signed-rank test (1000 permutations). Between-sample Bray-Curtis dissimilarities were calculated and principal coordinate analysis (PCoA) was performed and tested for significant differences between the two cohorts using PERMANOVA. During the beta diversity analysis we adjusted for DNA isolation batch and time in mail since these were the technical covariates. Linear regression models intending to determine significantly different genera between both cohorts were performed in MaAsLin. During analysis we adjusted for BMI, sex, technical covariates (TIM and Batch), and multiple testing by FDR (q < 0.05).

To compare the functional metagenome of gut bacteria between children and adults, we used the PICRUSt (v.1.1.0) tool^45^ to obtain predicted bacterial functions. HUMAnN2 (v0.99) was used to identify the Kyoto Encyclopedia of Genes and Genomes (KEGG) pathways^46^. For the identification of the specific pathway biomarkers distinguishing the gut microbiome of the children from those of adults, we performed a linear discriminant effect size (LEfSe) analysis^47^ with the default settings: α (ANOVA) = 0.05 and logarithmic LDA (linear discriminant analysis) score = 2.0.

## Results

### Stool microbiota 16S rRNA data generation in the GenR and RS cohorts

#### Selection of subjects

For GenR 4,959, and for RS 2,440 participants were invited to provide a stool sample. In total, 2,921 (response rate = 59%) and 1,691 (response rate = 69%) were received at Erasmus MC for GenR and RS stool samples, respectively (Supp. Fig. 1). Excluding antibiotic users resulted in exclusion of 196 samples from GenR and 7 samples from RS. In GenR, individuals who used antibiotics in the last year had a significantly lower microbiome diversity and altered microbiome composition (Supp. Table 1). For probiotic use and recent travelling activity outside the Netherlands we could not detect significant effects on diversity or composition in the two cohorts (Supp. Table 1). After quality control (Supp. Fig. 1), 16S rRNA data of 2,214 subjects in the initial dataset of GenR and 1,544 subjects in the initial dataset of RS were included.

#### Assessing the influence of technical co-variates and sample exclusion

Since stool samples were collected at ambient temperature via regular mail, we assessed the effects of time in the mail (TIM) on the microbiome profiles. Furthermore, since DNA-yields varied across different DNA isolation batches (Supp. Fig. 3), we assessed its effect on the microbiome profiles as well.

**Figure 1:**
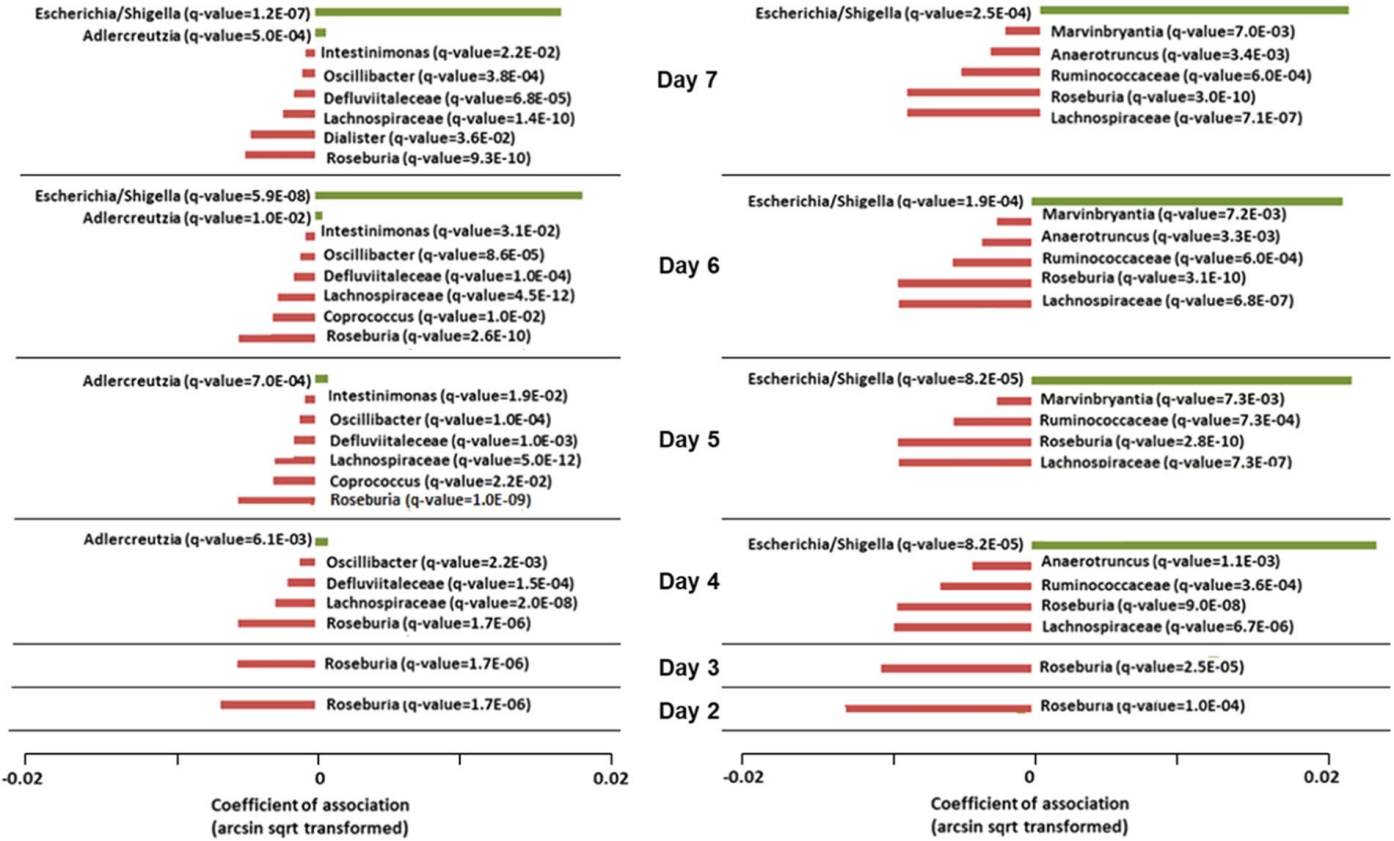
Effect of ambient temperature on individual OTUs. Regression analysis of individual OTUs with time in mail (TIM) for samples in GenR (**A**) and RS (**B**). At each TIM. the initial OTU table was sub-sampled to contain only samples up to that TIM. Red bars indicate bacteria that decreased in abundance and green bars bacteria that increased upon increasing TIM. Q-values are indicated; only significantly abundant OTUs are presented.

Upon longer periods of TIM (except for day 7 in GenR), an increase in the relative abundance of phylum Proteobacteria was observed in the average profiles of both cohorts (Supp. Fig. 4). For RS only, an increase in phylum Bacteroidetes was observed between days 6 and 7. In contrast, we did not observe any substantial changes in phylum-level profiles with respect to the DNA-Batch variable (Supp. Fig. 4). Moreover, TIM had a small but significant negative effect on α-diversity in GenR and RS (beta = −0.02 alpha units/day, p = 9.3e-03 and beta = −0.03, p = 4.6e-03, respectively). Again, we did not observe an effect of Batch on α-diversity. Correlations of TIM and Batch with overall composition (β-diversity) in both cohorts were small but significant (R^2^ = 0.004, p = 0.001 and R^2^ = 0.002, p = 0.005 for TIM and R^2^ = 0.01, p = 0.001 and R^2^ = 0.005, p = 0.001 for Batch in GenR and RS, respectively). In taxonomy-based analyses (MaAsLin) at genus-level of both datasets for TIM, we observed increased abundances of genus *Escherichia/Shigella* upon prolonged times in the mail (Fig. 1). However, these differences were only significant after 3 days in the RS cohort and after 5 days in the GenR cohort (Fig. 1). Furthermore, we observed smaller decreases in the abundances of a number of genera, including *Roseburia* and *Coprococcus*, upon prolonged TIM.

In order to further evaluate the importance of TIM and Batch effects, we added them to linear models for the analysis of well-established association of α-diversity with BMI^11,13,40^ in both GenR and RS. Although, the estimates (betas) of the exposure (i.e., BMI) remained similar after various levels of adjustment, the model including sex, age and TIM (model 1) as co-variates provided a better fit to the data than the model including only sex and age (model 0; likelihood ratio test: p = 5.8e-03 and p = 5.5e-03 for GenR and RS, respectively). Including Batch, within model 1 (model 2), resulted in a better fit to the data in RS only (Table 1; p = 1.9e-04).

**Table 1:** The effect of technical and biological covariates on the association of BMI with Shannon diversity in the 16S datasets of GenR and RS cohorts.

	Initial dataset	GenR (N = 2,214)			RS (N = 1,544)		
	Linear model: α-diversity ~ BMI + covariates	R^2^	Estimate	P-value	R^2^	Estimate	P-value
	BMI	0.008	−0.031	9.6e-06	0.015	−0.021	9.9e-07
	BMI + sex	0.008	−0.031	7.3e-06	0.014	−0.021	1.0e-06
Model 0	BMI + sex + age	0.008	−0.032	7.0e-06	0.015	−0.021	7.8e-07
Model 1	BMI + sex + age + TIM	0.011	−0.032	1.0e-05	0.019	−0.021	9.2e-07
Model 2	BMI + sex + age + TIM + Batch	0.011	−0.032	1.0e-05	0.027	−0.020	1.6e-06
	BMI + sex + age + TIM + Batch + ethnicity	0.038	−0.021	3.2e-03			

Stepwise linear model used for each covariates in the analysis of association of microbial diversity with BMI (TIM: time in mail).

Based on the results of the effects of the technical covariates on the profiles in the RS and GenR cohorts, only stool samples that arrived at the research center within 3 days for RS and within 5 days for GenR, were included in the final 16 S datasets (Fig. 1). Furthermore, TIM and Batch were included as technical covariates in all further analyses.

### Description and validation of final 16S rRNA datasets of the GenR and RS cohorts

After quality control and sample exclusion, the final 16 S datasets comprised 2,111 individuals in GenR and 1,427 subjects in RS. The average age of GenR children was 10 (range = 9 to 12) years and included participants from 13 self-reported ethnicities with Dutch being the most frequent one (62%; Table 2 and Supp. Fig. 5). The average age of RS participants in the final 16S dataset was 57 years (range = 46 to 88) and 82% were Dutch based on self-reported ethnicity (Table 2 and Supp. Fig. 5).

**Table 2:** Characteristics of GenR and RS cohort.

	GenR	RS
Total	2.111	1.427
Females (%)	50	58
Age (years ± SD)	9.8 ± 0.32	56.8 ± 5.9
BMI (kg/m² ± SD)	17.3 ± 2.4	27.5 ± 4.5

Age and BMI information at the time of visit at which the fecal sample was collected.

**Figure 2:**
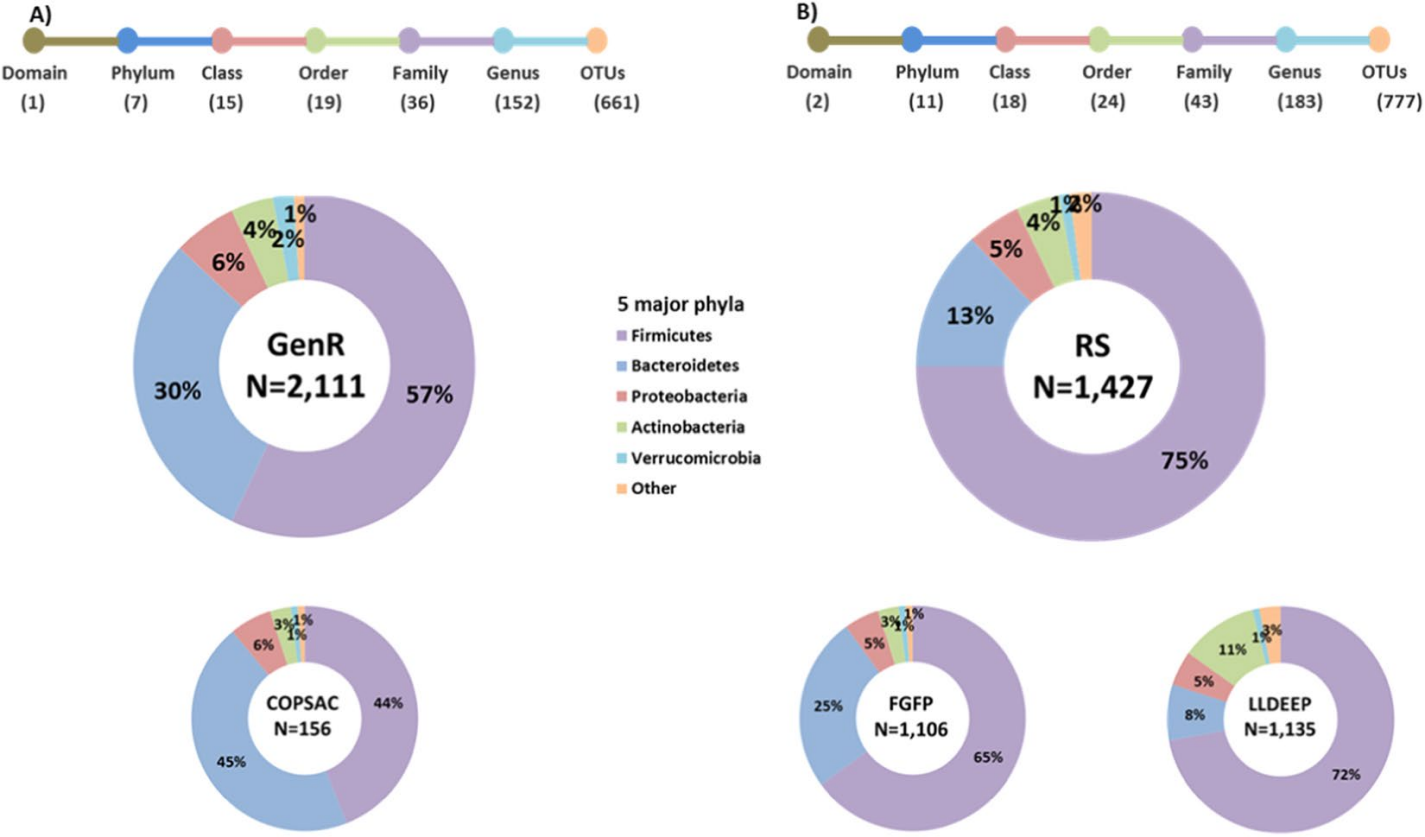
Characteristics of the final datasets of the two cohorts. GenR (**A**) and RS (**B**). Number of observed taxa at each taxonomy level Top: indicates the number of unique OTUs identified in each taxonomic clade, top A: RS cohort, top B: GenR cohort. Bottom: Donut plots indicate the average relative abundances of the top major phyla in each cohort. Donut plots of the COPSAC cohort (children aged 6 years) and doughnut plots of FGFP and LLD cohorts (adults) are plotted for comparison with the abundance in GenR and RS.

A total number of 661 OTUs were identified in the GenR cohort and 777 OTUs in the RS cohort (Fig. 2). Of these, 656 OTUs overlapped between the two cohorts, leaving 5 OTUs specific for GenR and 42 OTUs specific for RS. For both datasets, variations in overall microbiome profiles were driven by the relative abundances of the four major phyla Firmicutes, Bacteroidetes, Proteobacteria and Actinobacteria (Supp. Fig. 6). We observed higher Shannon α-diversities in RS (mean 4.02; SD = 0.50) than in the GenR cohort (mean 3.81; SD = 0.57; Fig. 2). Furthermore, we observed the absence of kingdom Archaea and a lower abundance of phylum Firmicutes in the GenR cohort as compared to RS (Fig. 2).

To validate our datasets, we compared the averaged phylum level profiles of our cohorts with those of other cohorts of similar characteristics (i.e., age range, ethnic background). In addition, we tested the well-established association between BMI and alpha diversity^11,13,40^, and we determined the association between single OTUs and BMI and compared these results with other cohorts.

To compare GenR and RS 16S datasets with other populations, we used the 16S datasets of the COPSAC^39^, LLD^40,41^ and FGFP^42^ cohorts. The average phylum-level profiles of GenR were similar to those of COPSAC, and the average phylum-level profiles of RS were similar to those of LLD and FGFP (Fig. 2). The lower abundance of phylum Firmicutes in the GenR cohort was also observed in the COPSAC cohort^39^.

Further, in order to validate our microbiome datasets, we investigated the association of gut microbiome with BMI. We excluded subjects of non-Northern European origin from both GenR and RS, which resulted in a dataset of 1,712 GenR samples and a dataset of 1,371 RS samples.

α-diversity was negatively associated with BMI after adjusting for age, sex and technical covariates in both cohorts (beta = −0.019, SE = 2.3e-03, p = 1.1e-03 for GenR and beta = −0.015, SE = 2.8e-03, p = 1.8e-06 for RS). We compared this association with observations reported in similar  cohorts of Northern European origin (LLD and FGFP). The negative correlation of α-diversity with BMI was in line with findings reported previously^40^. At genus-level, we identified 27 BMI-associated genera in GenR and 33 BMI-associated genera in RS (Table 3, Supp. Table 2), of which 14 genera overlapped across both cohorts. In the RS cohort, we confirmed 6 genera found to be associated with BMI in the LLD and FGFP cohorts (Table 3). In GenR we only confirmed two genera (*Alistipes* and *Barnesiella*) to be associated with BMI in RS, LLD and FGFP cohorts. As previously reported^40,48^ we also observed the association between increased abundance of genus *Akkermansia* and lower BMI.

**Table 3:** Single OTU associations with BMI in the GenR and RS cohorts and further replication in FGFP and LLD.

Genus	GenR		RS		FGFP		LLD	
	N = 1,712		N = 1,371		N = 1,106		N = 1,135	
	Coefficient	q-value	Coefficient	q-value	Coefficient	q-value	Coefficient	q-value
*ChristensenellaceaeR7group*	−0.0044	8.3E-04	−0.0034	6.5E-08				
*Alistipes*	−0.0024	8.1E-03	−0.0015	4.2E-04	−0.1326	9.7E-06	−0.0026	4.3E-05
*Ruminococcus1*			−0.0014	2.5E-03				
*Coprococcus2*			−0.0014	1.7E-02				
*Ruminiclostridium6*	−0.0019	3.4E-04	−0.0012	1.1E-03				
*Anaerotruncus*			−0.0008	1.0E-03			−0.0016	8.5E-02
*Barnesiella*	−0.0016	9.2E-03	−0.0007	2.9E-03	−0.0761	1.1E-02	−0.0030	5.3E-02
*Akkermansia*			−0.0006	3.8E-02				
*Ruminiclostridium9*	−0.0005	6.4E-03	−0.0004	4.3E-03				
*Odoribacter*	−0.0010	1.6E-03	−0.0004	1.1E-02			−0.0019	8.1E-02
*Oscillospira*			−0.0004	2.4E-03	−0.0933	1.9E-03		
*Butyricicoccus*			0.0006	1.2E-02				
*Lactobacillus*			0.0006	8.5E-04				
*Lachnoclostridium*			0.0006	4.5E-02				
*Coprococcus3*			0.0008	3.3E-02				
*Dorea*			0.0014	2.3E-03	0.1408	2.6E-06		
*Blautia*			0.0021	4.6E-02				
*Streptococcus*			0.0026	1.1E-05				
*Parabacteroides*	−0.0015	2.6E-02						
*Oscillibacter*	−0.0005	3.1E-02						
*Terrisporobacter*	0.0005	4.2E-02						
*Intestinibacter*	0.0014	9.7E-04						
*Romboutsia*	0.0020	2.6E-03						
*Bifidobacterium*	0.0046	3.2E-04						

Bacterial associations with BMI in the RS, GenR and other cohort studies. The +/− sign of the coefficient values indicate the direction of the correlation of the genus with BMI. q-value = FDR corrected P-value. Only known bacteria are presented.

### Comparison of RS and GenR stool microbiome diversities, compositions and functions

**Figure 3:**
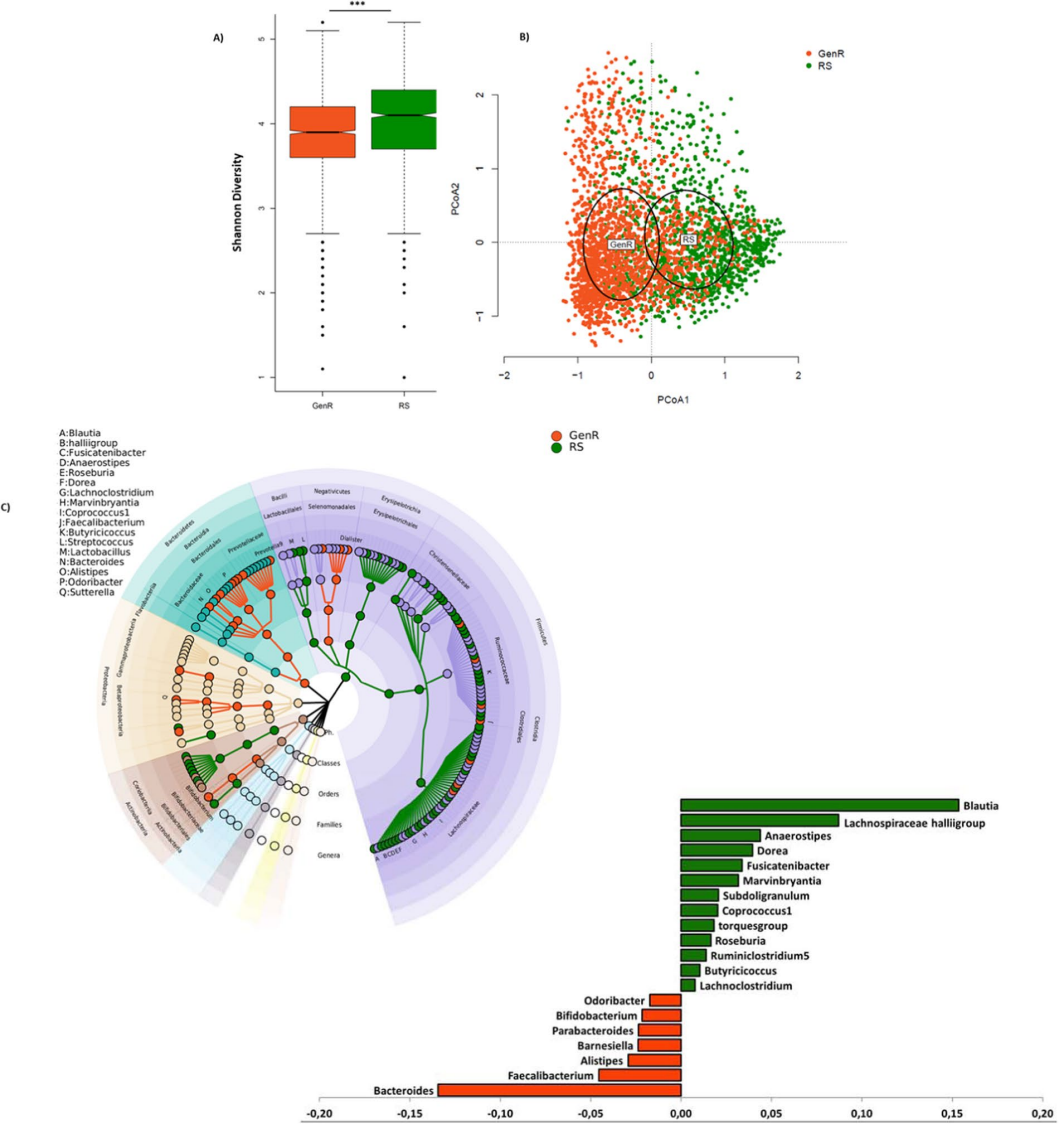
Comparison of the gut microbiome diversity and composition between adults (RS) and children (GenR). (**A**) boxplots of the Shannon diversity Index. (**B**) ordination plot of the gut microbiome composition in the two cohorts based on Bray-Curtis dissimilarities. The centroid and dispersion of each cohort is represented by the cohort name and ellipses, respectively. Clustering of RS and GenR was tested for significance using PERMANOVA. (**C**) Circular representation of the taxonomic tree of the microbiome compositions of the two cohorts. Each node represents one taxon at different taxonomic level. Orange nodes are the taxa that were observed with higher abundance in the GenR cohort and green nodes represent the taxa that were higher abundant in the RS cohort. (**D**) The genera represented the most in each cohort. On the x-axis the arcsine squared root transformed coefficients of the most significantly abundant genera in each cohort are shown. Orange bars represent GenR and green bars represent RS. Minus signs in the x-axis are used only for visualization.

To analyze differences in gut microbiome compositions between children and adults, both datasets were combined after exclusion of the non-Northern European samples. The combined dataset included 3,082 samples: 1,371 from RS and 1,712 from GenR and the final OTU table contained 173 genera. Although Shannon α-diversity was not associated with age in each cohort separately (linear model; GenR: beta = −0.003, p = 0.91; RS: beta = 0.001, p = 0.63), Shannon α-diversity was significantly higher in the RS cohort than in the GenR cohort in the combined dataset (p < 2.2e-16, mean = 5.9, sd = 0.74 for RS and mean = 5.58, sd = 0.82 for GenR). Also, the overall compositions (Bray Curtis distances) differed significantly between both groups (Fig. 3B; PERMANOVA; R^2^ = 0.06; p = 0.001). We observed a 2 to 3 times lower abundance of phylum Firmicutes in the GenR cohort as compared with RS (p < 2.2e-16). Furthermore, we observed higher abundances of the gram-negative classes (Bacteroidia, Negativicutes and some classes from phylum Proteobacteria) in children. Regression analysis, in MaAsLin, on the relative abundances of the individual genera showed higher relative abundances of 59 genera in RS compared to GenR and higher relative abundances of 20 genera in GenR compared to the RS cohort (q < 0.05; Supp. Table 3). The largest differences were observed for genera from family Lachnospiraceae including *Blautia, Lachnospiraceae, Anaerostipes, Dorea, Fusicatenibacter, Coprococcus, Roseburia, Ruminoclosteridium, Butyricicoccus* and *Lachnoclostridium* that were more abundant in RS, and genera from families Ruminococcaceae and Bacteroidaceae including *Bateroides, Faecalibacterium, Alistipes, Barnesiella, Parabacteroides, Bifidobacterium* and *Odoribacter* that were more abundant in GenR (Fig. 3C). The most abundant genera were *Bacteroides* in GenR (children) cohort and *Blautia* in the RS (adults) cohort (Fig. 3D).

**Figure 4:**
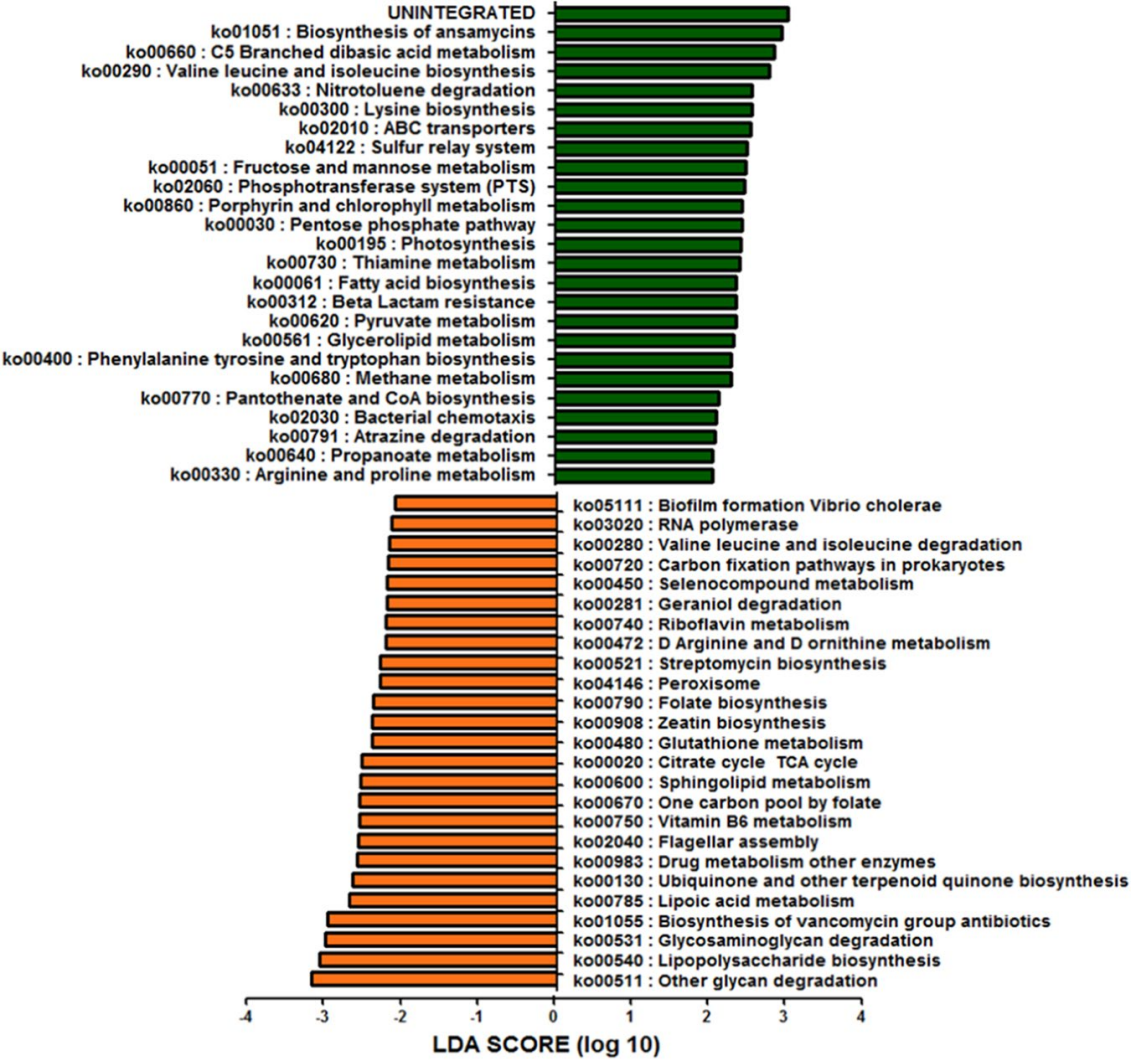
Predicted functional composition of metagenomes based on 16S rRNA gene sequencing data from GenR and RS cohorts. LEfSe based on the PICRUSt dataset revealed differentially enriched metabolic pathways associated with GenR (orange) or RS (green).

To assess functional differences in the gut microbiome between children and adults, we predicted the functional content based on the 16S rRNA data using PICRUSt. Analyses of the differences in the predicted functional metagenomics data between children and adults showed significant overrepresentation of 25 pathways in children and 25 pathways in adults (Fig. 4, Supp. Table 4). A remarkable difference between GenR and RS was the predominance of catabolic pathways in GenR (Val, Leucine, iso-Leucine degradation; ko00280) and its opposite in RS (Val, Leucine, iso-Leucine biosynthesis; ko00290). Putative colonization-related pathways like biofilm formation (ko05111), flagellar assembly (ko02040) and LPS biosynthesis (ko00540) were only enriched in GenR cohort.

## Discussion

In this publication we report on the 16S stool microbiota profiles of 3,538 subjects from two large, deeply phenotyped and well-characterized population-based cohorts: the Generation R (GenR) Study and the Rotterdam Study (RS). The 16S microbiome datasets originated from both children (GenR: n = 2,111) and adults (RS: n = 1,427) populations.

In general, the stool microbiota within RS had similar profiles as profiles in the previously published LLD and FGFP cohorts^40–42^. Similarly, our GenR stool microbiota possessed similar profiles as those observed in the COPSAC cohort^39^.

Further, both of our cohorts replicated the previously reported negative association between α-diversity and BMI^40^. Additionally, BMI was also associated with community composition (Bray-Curtis) in both RS and GenR cohorts, as reported for the LLD and FGFP cohorts. Also, *Alistipes* and *Barnesiella* were reported as negatively associated with BMI in all four of these cohorts. Interestingly, the lower Firmicutes abundance observed in the GenR and COPSAC children cohorts compared to RS, LLD and FGFP adult cohorts suggests an age-specific phenomenon is responsible for this difference. However, understanding these differences merit further investigation. Thus, as both our RS and GenR datasets contain many characteristics that are similar to previously published large cohort studies, we conclude that our datasets allow valid investigations into the composition and variation of the gut microbiota across child and adult subjects.

Comparing the gut microbiome composition between children and adults in a combined dataset of 3,083 children and adults showed significant clustering of the cohorts based on overall composition (PCoA beta diversity) and significant different α-diversities. The lower diversity in GenR suggested a lack of ‘maturation’ of the gut microbiome in children. However, we did not observe a significant age-related change in alpha diversity within each cohort separately. This might be due to the narrow age-range in both cohorts (9.8 ± 0.32 in GenR, 56.8 ± 5.9 in RS, Table 2). At genus level, the relative abundances of genus *Bacteroides* were higher in GenR than in RS. Members of the genus *Bacteroides* are specialized at utilizing both plant and host-derived polysaccharides^49–52^. As compared to other genera, *Bacteroides* have a large number of genes specialized to metabolize various glycans. They also have environmental sensors that control their expression upon exposure to glycans. The predicted metagenomics data showed indeed higher abundances of glycan degradation pathways in children’s gut microbiome than in adults. In contrast, the relative abundances of the genus *Blautia* were higher in RS than in GenR. *Blautia* digest complex carbohydrates like whole-grains^53^ and its abundance has been shown to be reduced in patients with colorectal cancer^54^ and in children with type 1 diabetes^55^. Its abundance has also been reported to be inversely related to bone mineral density in human^56^. In fact, the relative abundance of *Blautia* and *Bacteroides*, has been implicated as a determinant of the so-called ‘healthy microbiome’ due to differences in the metabolic functions of these genera^54,55,57,58^.

With respect to metabolic functions, we identified many predicted carbohydrate pathways that were significantly enriched in adult versus child metagenome datasets, with many of these carbohydrate pathways involving the metabolism of simple sugars such as fructose and mannose. However, whether these differences in microbiota profiles can be explained by possible differences in energy demand and metabolic programming between adults and children remains to be proven.

Other interesting differences in the predicted functional pathways between children and adults, were observed in antibiotics synthesis pathways. The vancomycin biosynthesis pathways were significantly higher in the gut microbiome of children as compared to adults. Vancomycin is a glycopeptide with activity against gram-positive bacteria, while gram-negative bacteria are resistant to this drug because of their distinct cell-wall^59,60^. On the other hand, biosynthesis of ansamycin was higher in adults than in children. Ansamycin is another antibiotic with activity against gram-positive, as well as gram-negative bacteria^61^. These findings indicate potential differential anti-bacterial activities between the microbiota of children as compared to adults, which appears to be in line with our observation of higher relative abundances of gram-negative bacteria in the gut microbiota of children. However, the exact role of antibiotic production in modulating the microbiota profiles of children and adults remains to be shown.

Another difference identified by predictive functional pathway analysis was the differential biosynthesis of vitamin B classes by the gut microbiota of children and adults. Children’s gut microbiomes are associated with pathways related to increased biosynthesis of vitamins B2, B6, and B9 (folate), while in adults vitamin B1 and B5 biosynthesis pathways appear to be increased. This predictive finding could be linked to age-dependent differences in, for example, the requirement for folate, which is particularly important for cell division and growth during pregnancy and childhood.

In children, the enrichment of putative colonization-related pathways involving biofilm formation, flagella assembly and LPS biosynthesis, may provide insights into the development of intra-microbiome and host-microbiome interactions, and also be linked to our observation of higher relative abundances of gram-negative bacteria in children - LPS is a major constituent of the cell wall of gram-negative bacteria.

Finally, observing photosynthesis pathway (ko00195) in the functional data could be explained by the presence of Cyanobacteria in our datasets, as Cyanobacteria are photosynthetic bacteria. Likely, the photosynthesis pathways detected in the PICRUSt analysis were derived from these Cyanobacteria. The presence of Cyanobacteria in the gut may have a dietary origin^62^.

As with all studies, the current study was not free of limitations. One of the main discussion points involves the fact that stool samples were not directly frozen and immediately stored at −80 °C. For logistical reasons relating to the large scale cohorts recruited, we depended on postal delivery of home-collected stool samples to the research laboratory. Several studies have addressed the effects on microbiota composition when sample collection is performed at room temperature^63–68^, and observed that longer periods of storage at ambient temperature may affect microbiota profile composition and decrease diversity in the samples. We, therefore, analyzed the effects of stool sample collection at ambient temperature in both cohorts. Within our own initial datasets, we addressed this issue by using several approaches and observed increased abundances of *Escherichia/Shigella* when stool samples were mailed to the research laboratory and received after 3 days (RS) or after 5 days (GenR) in the post. Therefore, to avoid the influence of any delayed processing time, we excluded samples that had been in the mail for longer than 3 days in RS and 5 days in GenR cohorts.

Next to the increase in abundance of *Escherichia/Shigella* upon prolonged times in the mail, we observed several taxa that decreased over time (*Coprococcus* and *Roseburia*). These decreases were, however, relatively low and likely a consequence of the compositionality of the data: if one OTU increases, other OTUs decrease. We decided to adjust for these technical artifacts in further analyses by including time in mail as a technical cofactor.

The actual explanation for the difference in microbiota composition stability over time between RS and GenR cohorts is likely due to the fact that GenR participants were asked to keep their samples in their home fridge at 4 °C (for posting on Monday) if they were produced at the weekend, allowing better preservation of samples compared to RS participants who mailed their samples over the weekend. As well as the exclusion of 3 day and 5 day samples, we also included TIM as a technical covariate in all analyses.

Next to excluding samples that had been in the mail for too long, we also excluded recent antibiotic users from our datasets (196 samples from GenR and 7 samples from RS). Antibiotic use had a significant effect on alpha diversity and overall composition in GenR (Supplementary Table 1), whereas in RS the number of users was too small to have a detectable effect. Probiotic use and travel abroad were also recorded, but we could not detect significant effects on alpha diversity and composition in both cohorts. Given the small effect size of probiotic use, this study might be underpowered to conclude a lack of effect.

Recent studies indicate that the method of DNA isolation is the main source of technical variance in microbiome studies^69–71^. We, therefore, analyzed the effect of DNA isolation throughout the 391 runs (134 runs for RS and 257 runs for GenR) that were performed in this study and observed a batch effect causing a reduction in average DNA yield per sample in a proportion of the hundreds of runs performed. As we could not trace any clear cause for this technical artifact we introduced a “Batch” variable that allowed us to discriminate between low yield and the high yield runs. This “Batch” variable was significantly associated with overall profiles in the GenR and RS cohorts and was included as technical covariate in all analyses. Another, more general limitation when comparing different cohort datasets, is the accuracy of replication of 16S profiles, since sample collection, sample storage, DNA isolation, PCR amplification, amplified 16S rRNA variable region, the sequencing technique used and downstream bioinformatics may differ between different cohorts. For example, out of 33 associated OTUs with BMI, we could only replicate 6 in LLD and FGFP. In addition, geographical differences and lack of repetition may limit the accuracy of replicating microbiota profiles from different countries. In addition, although (besides using harmonized sample collection and dataset generation) we adjusted for the potential confounders in the analyses comparing children and adults, we should be aware that we cannot totally discard the presence of residual stratification as a consequence of the fact that both populations were derived from different cohorts.

To conclude, in this study we performed microbiota profiling on the stools of 2,111 children in the age-range of 9 to 12 years and 1,427 adult individuals in the range of 46 to 88 years of age. We observed a clear distinction between the gut microbiomes of children as compared to adults, including differences in microbiota diversity and Firmicutes and Bacteroidetes abundances. These changes were associated with predicted shifts in functional properties, including in energy metabolism, antibiotic production and the production of essential B-vitamins. These observations are likely due to the development of human-gut microbiome interactions with age. As both GenR and the RS cohort have been deeply characterized^24,25^ we here presented two valuable datasets for studying the possible association of the human stool microbiome and, life style, environmental factors, and health and disease outcomes. In addition, as the participants of both cohorts have been genotypes, also association between host genetics and host microbiome could be investigated.

URLs. The Rotterdam Study: http://www.ergo-onderzoek.nl/wp/; Generation R study: https://www.generationr.nl/researchers/; QIIME: http://qiime.org/; USEARCH: https://www.drive5.com/usearch/; PEAR: https://sco.h-its.org/exelixis/web/software/pear/; TAGcleaner: http://tagcleaner.sourceforge.net/; Vegan R package: https://cran.r-project.org/web/packages/vegan/index.html; phyloseq: https://joey711.github.io/phyloseq/; PICRUSt: http://picrust.github.io/picrust/; HUMAnN2: http://huttenhower.sph.harvard.edu/humann2; LEfSe: https://bitbucket.org/biobakery/biobakery/wiki/lefse.

## Supplementary information

Supplementary figures.

Supplementary tables.

## Data Availability

All relevant summary data supporting this study are available within the article and its supplementary information files, or additional unpublished data can be provided by the corresponding author upon reasonable request. Due to ethical and legal restrictions, individual-level data of the Generation R Study and Rotterdam Study cannot be made publicly available. These data are available upon request to the data manager of the Rotterdam Study Frank van Rooij (f.vanrooij@erasmusmc.nl) or of the Generation R Study Claudia Kruithof (c.kruithof@erasmusmc.nl) and subject to local rules and regulations. This includes submitting a proposal to the management team of RS, where upon approval, analysis needs to be done on a local server with protected access, complying with GDPR regulations.
