# Supplementary material for: Diversity, compositional and functional differences between gut microbiota of children and adults
Source: Sci Rep. 2020 Jan 23;10:1040. doi: 10.1038/s41598-020-57734-z (PMC6978381; doi:10.1038/s41598-020-57734-z)
Supplement: Supplementary file 1 — Supplementary figures. [file 41598_2020_57734_MOESM1_ESM.docx]

**Diversity, compositional and functional differences between gut microbiota of children and adults**

Djawad Radjabzadeh^1^, Cindy G. Boer^1^, Sanne A. Beth^2,3^, Pelle van der Wal^1^, Jessica C. Kiefte-De Jong^2,3,6,7^, Michelle A.E. Jansen^2^, Sergey R. Konstantinov^4^, Maikel P. Peppelenbosch^4^, John P. Hays^5^, Vincent W.V. Jaddoe^3,6^, M. Arfan Ikram^6^, Fernando Rivadeneira^1,3,6^, Joyce B.J. van Meurs^1,6^, André G. Uitterlinden^1,3,6^, Carolina Medina-Gomez^1,3,6^, Henriette A. Moll^2^, Robert Kraaij^1*^

^1^Department of Internal Medicine, ^2^Department of Paediatrics, ^3^The Generation R Study ^4^Department of Gastroenterology and Hepatology, ^5^Department of Medical Microbiology and Infectious Diseases, ^6^Department of Epidemiology, Erasmus MC, University Medical Centre Rotterdam, Rotterdam, the Netherlands, ^7^Department of Public Health and Primary Care/LUMC Campus The Hague, Leiden University Medical Centre, Leiden, The Netherlands

*Corresponding author: Erasmus MC, Room Ee579, P.O. Box 2040, 3000 CA Rotterdam, The Netherlands. r.kraaij@erasmusmc.nl

**Keywords**

Stool microbiota, 16S rRNA profiling, population-based cohort study, Rotterdam Study, Generation R Study, children, adults

**Supplementary Information**

**
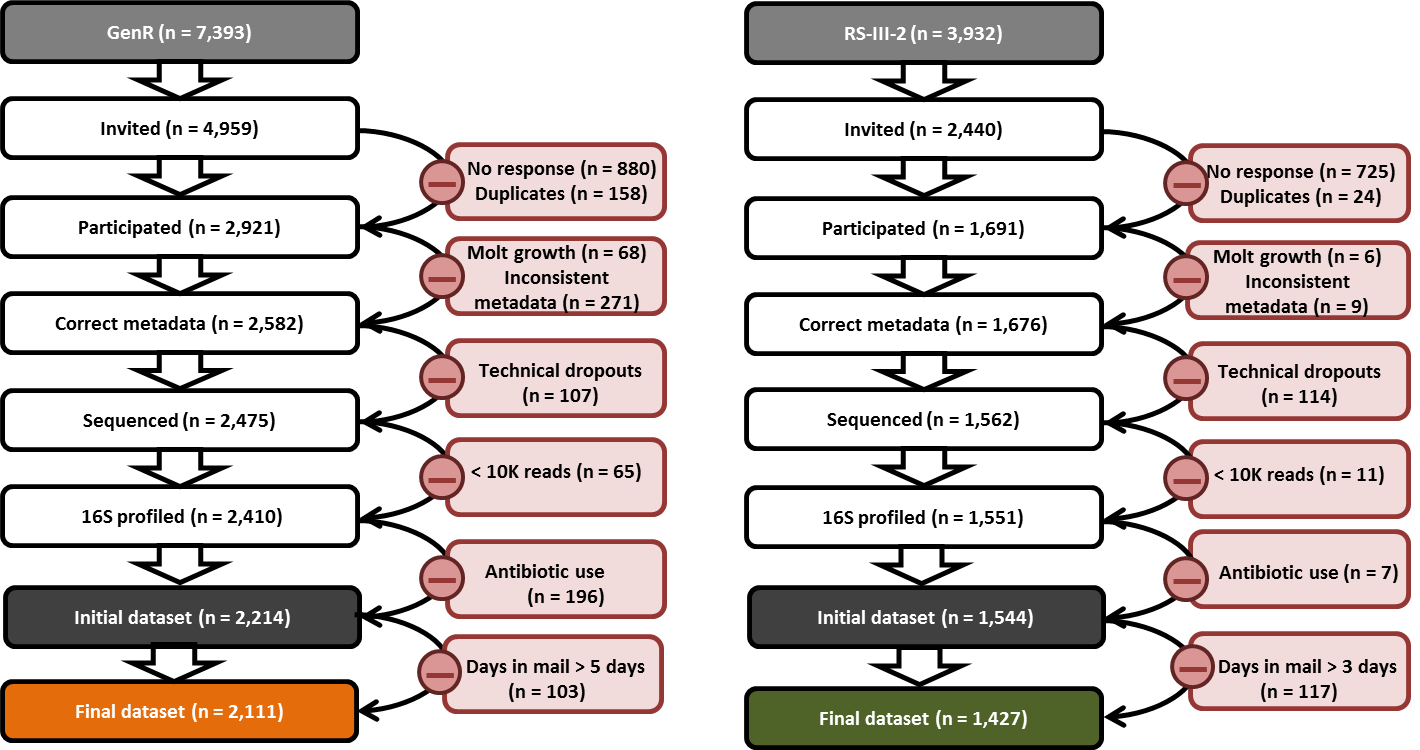
**

**Supplementary Figure 1.** Overview of sample inclusion, exclusion and dropout at each step. Light grey rectangles are the starting cohorts, and white rectangles are the number of samples remained after each exclusion step. The dark grey rectangles are the initial datasets, and the orange rectangle represents the final dataset of the GenR cohort and the green rectangle represents the final dataset of the RS cohort.


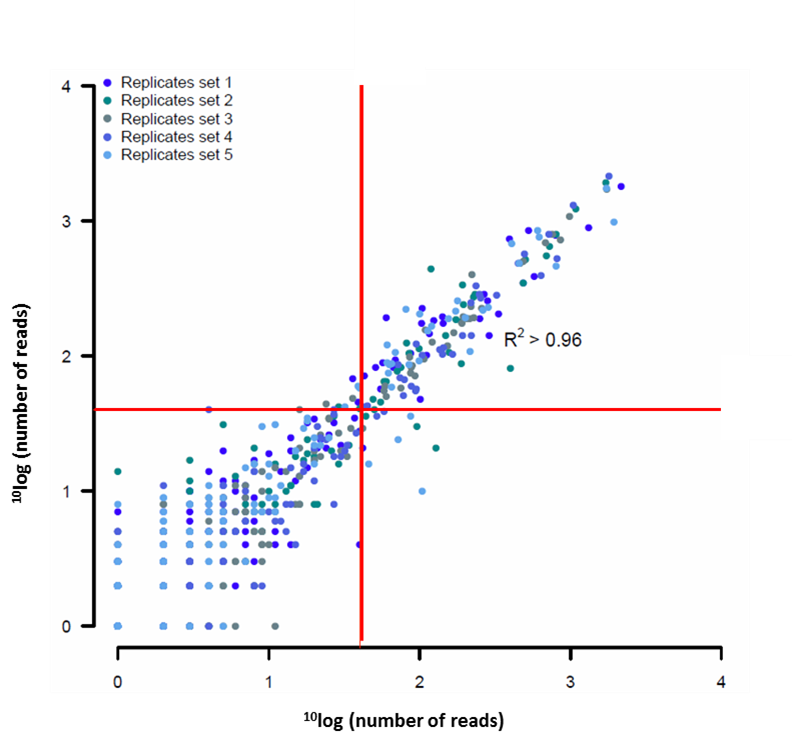


**Supplementary Figure 2.** Correlation of absolute counts of OTU abundances of 5 technical replicates. Stool DNA from 5 individuals were amplified for V3-V4 hypervariable regions of the 16S rRNA gene twice and sequenced twice. The spearman correlation was calculated between the log-transformed obtained OTU tables per individual. Spearman correlation exceeded 0.96 above 40 reads per OTU (red-lines).


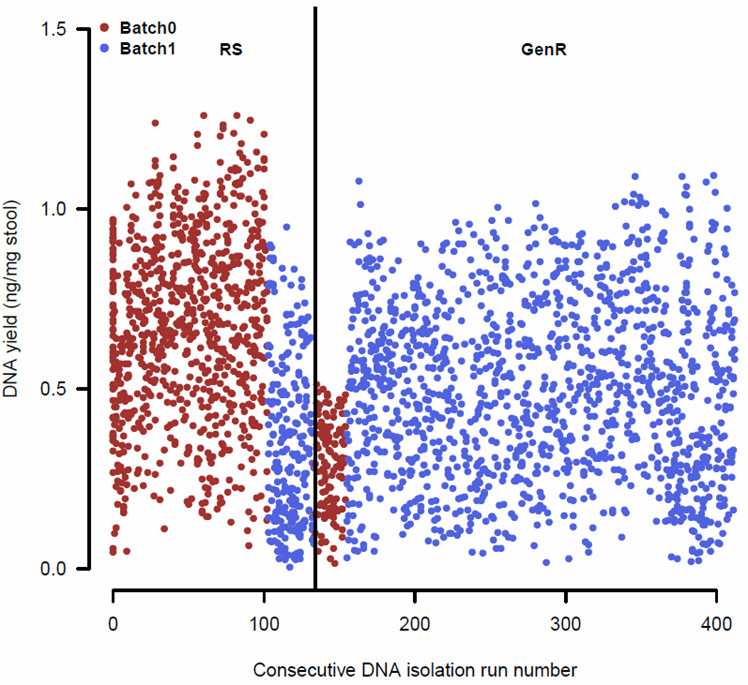


**Supplementary Figure 3.** Batch effect of the DNA isolation runs. DNA yields of individual samples per consecutive DNA isolation run in batches of 12 samples. Batches per cohort are represented by different colors (red and blue); cohorts are separated by the vertical black line.


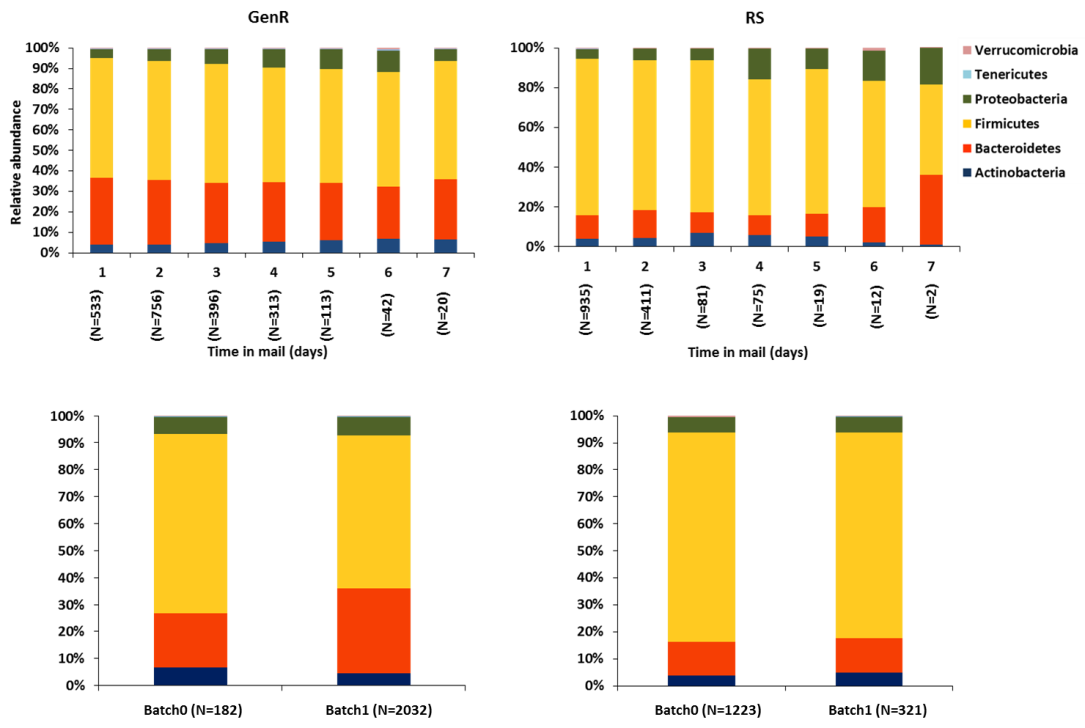


**Supplementary Figure 4.** Average profiles based on the 6 major phyla per time in the mail (TIM in days) for the GenR (top left panel) and RS (top right panel) cohorts. Bottom panels represent the average profiles based on the 6 major phyla per DNA isolation batch (Batch) in GenR (left) and RS (right).


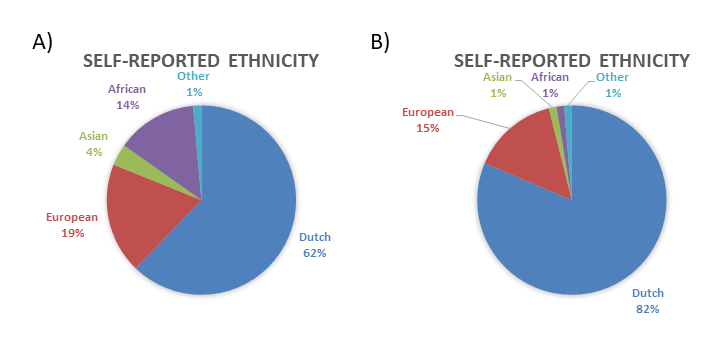


**Supplementary Figure 5.** Distribution of self-reported ethnicities in the GenR (A) and RS (B) cohorts.


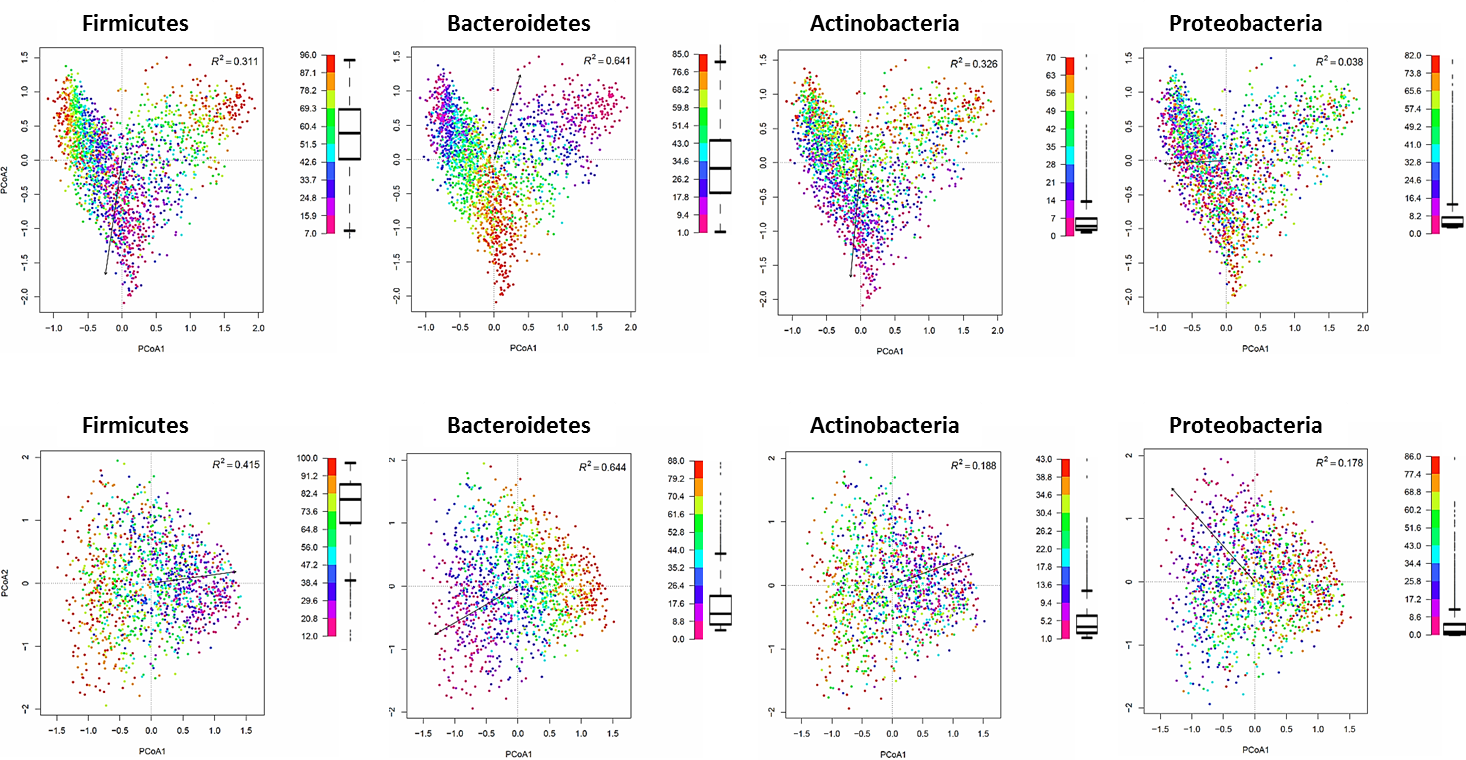


**Supplementary Figure 6.** Ordination plots of the microbiome datasets of GenR and RS cohorts. Top panel: GenR and bottom panel: RS. The abundances of the 4 major phyla (Firmicutes, Bacteroidetes, Actinobacteria and Proteobacteria) are colored from high to low.
